# Prevalence and Associated Risk Factors of Intestinal Parasitic Infections Among Patients Requesting Stool Examination at Kidus Harvey Health Center, Ayna Bugina District, North Wollo, Ethiopia

**DOI:** 10.1155/japr/5596158

**Published:** 2025-04-23

**Authors:** Awoke Eshetie, Tilahun Yohannes, Muluken Dejen

**Affiliations:** Department of Biology, College of Natural and Computational Sciences, University of Gondar, Gondar, Ethiopia

**Keywords:** Ayna Bugina, health center, intestinal parasite, prevalence, risk factor

## Abstract

Intestinal parasitic infections (IPIs) remain a significant global health burden, disproportionately affecting developing nations. This cross-sectional study aimed to determine the prevalence and associated risk factors of IPIs among patients visiting Kidus Harvey Health Center in North Wollo, Ethiopia, from February to April 2023. Stool samples and questionnaire data were collected from 404 voluntary participants. Data were analyzed using SPSS version 20, employing descriptive statistics and logistic regression. The overall prevalence of IPIs was 41.09% (166/404). *E. histolytica/dispar* (16.34%) and *A. lumbricoides* (7.2%) were the predominant parasitic species. Significant risk factors identified included marital status (married: AOR = 3.536, 95%CI = 1.515–8.250, *p* = 0.003), occupation (farmers: AOR = 2.447, 95%CI = 0.816–7.337, *p* = 0.04), family size (> 9 members: AOR = 2.860, 95%CI = 0.619–13.206, *p* = 0.008), infrequent handwashing before meals, infrequent shoe wearing, contact with water bodies, untreated water sources, and raw meat consumption. The high prevalence of IPIs underscores the need for targeted public health interventions. These interventions should prioritize promoting personal and environmental sanitation, reducing raw meat consumption, and preventing unprotected contact with soil and water. Comprehensive public health campaigns delivering specific hygiene education to high-risk groups, emphasizing handwashing, shoe wearing, and safe water practices, are essential to mitigate the burden of IPIs in this population.

## 1. Background

Intestinal parasitic infections (IPIs) caused by helminths and protozoa remain a significant global health burden, particularly in developing countries [1, 2]. While both groups of parasites pose substantial morbidity and mortality burden on poor society [3–5], protozoans, including *Giardia lamblia* and *Entamoeba histolytica/dispar*, are more prevalent than helminths in endemic regions [6, 7]. The most common intestinal helminthes are *Ascaris lumbricoides*, hookworms, *Schistosoma mansoni*, and *Hymenolepis nana* [2]. Intestinal protozoa are primarily transmitted via the fecal–oral route through the ingestion of dormant cysts in contaminated food, water, or hands [8]. In contrast, most helminthic intestinal parasites are transmitted through the ingestion of eggs passed in the feces of an infected individual [9], the larvae of geohelminths actively penetrating the skin upon contact with contaminated soil [8].

Intestinal parasites pose a significant public health burden. These parasites can lead to severe health complications, including nutritional deficiencies, anemia, and impaired growth and development, especially in children [10, 11]. Key risk factors for IPIs are poor hygiene and sanitation, scarcity of potable water, unsafe human waste disposal systems and open field defecation, contamination, favorable environmental conditions for parasites, lack of adequate health services, and low level of awareness [12]. While previous studies have examined IPI prevalence in Ethiopia, there remains a dearth of epidemiological data for certain regions [6]. During the researcher's visit to the study area, it was observed that many individuals walked barefoot, and there were subpar personal and environmental hygiene practices, along with limited latrine coverage and inadequate waste disposal. These conditions suggest a high likelihood of IPI prevalence. Therefore, this study aimed to investigate the prevalence and associated risk factors of IPIs among patients seeking stool examinations at Kidus Harvey Health Center in Bugina District, North Wollo Zone, Ethiopia.

## 2. Materials and Methods

### 2.1. Description of the Study Area

Bugina District is situated in the North Wollo Zone of the Amhara Regional State, Ethiopia. Its administrative center, Ayna, is located approximately 754 km north of Addis Ababa. Based on the district's administrative office, the total population of Bugina District is estimated at 103,079, comprising 48,049 males (46.6%) and 55,030 females (53.4%). The study area is geographically situated at 12° 20⁣′ north latitude and 38° 45⁣′ east longitude. The primary economic activity of the local population within the study area is agriculture.

### 2.2. Study Design

A cross-sectional study was conducted from February to April 2023 to determine the prevalence of IPI and associated risk factors in the Kidus Harvey Health Center in the Ayna Bugina District, North Wollo, Ethiopia.

### 2.3. Study Population

The study population was people who visited Kidus Harvey Health Center for IPI diagnosis and treatment during the study period.

### 2.4. Inclusion Criteria

The included participants were individuals who visited the health facility for IPI diagnosis and treatment during the data collection period, provided informed consent, and reported no antiparasitic drug use within the preceding 2 months.

### 2.5. Sample Size Determination and Sampling Technique

#### 2.5.1. Sample Size Determination

The required sample size (*n*) was determined using a single population proportion formula for cross-sectional study as it was described in Naing et al. [13]

 $$\begin{array}{c} n=\frac{z^{2}*p\;\left(1-p\right)}{d^{2}}, \end{array}$$

where *n* = sample size, *z* = standard value, *P* = prevalence 50% (estimated prevalence), *d* = marginal error in the 95% confidence interval, *Z* = 1.96 and *d* = 5%. Therefore, the required sample size (*n*) was then calculated to be 384. To compensate for the nonrespondents and to minimize errors arising from noncompliance, 5% of the sample size was added; the final sample size was 404 study participants. A purposive systematic sampling technique was employed to select participants. Specifically, one individual was chosen from every three consecutive patients.

### 2.6. Data Collection Methods

#### 2.6.1. Collection of Sociodemographic and Risk Factor Data

A questionnaire survey was conducted to determine the main sociodemographic and potential risk factors for IPI among study participants. These questionnaires were initially developed in English and subsequently translated into local language, Amharic, to facilitate participant comprehension. The questionnaire was self-administered. Participants filled it out in their mother tongue. During the data collection process, the data collector meticulously observed and verified the participants' fingernail status (trimming of fingernails), overall hygiene practices, and footwear conditions.

#### 2.6.2. Collection of Stool Samples and Parasitological Examination

Following the acquisition of informed consent, participants were instructed on the proper collection of adequate, contamination-free stool samples. Each participant was provided with a labeled, disposable plastic cup and applicator stick and advised to collect approximately 5 g of stool, equivalent to the size of two matchsticks. Fresh stool samples were collected from all consenting participants and assigned unique codes. These samples were then examined by a laboratory technician at the parasitological laboratory unit of the health center.

The collected stool sample was first examined macroscopically to observe the presence of abnormal conditions such as unusual color, consistency, form, odor, and presence of mucus. The fresh stool was then prepared and diagnosed with direct wet mount and then formal ether concentration techniques. The prepared slides were investigated under light microscope with 10x and 40x objective lenses to detect the life stages of intestinal parasites. All slide preparation and examination were carried out according to the WHO guideline [14]. The diagnostic methods implemented in this study, wet mount and formol–ether concentration, have limitations. Wet mounts, while detecting motile parasites, risk missed detections due to low sensitivity and rapid sample decay. Formol–ether concentration may distort some cysts and is not ideal for all parasite types.

### 2.7. Data Quality Control

A structured questionnaire was developed in English, translated into Amharic (the local language), and then back-translated into English to ensure accuracy of meaning. To pilot test the questionnaire, it was administered to 10% of inpatients for IPI in the health center prior to data collection. However, the data were not included in the final analysis.

### 2.8. Data Analysis

The data from questionnaires and laboratory results were verified for accuracy and consistency and then coded and entered into SPSS version 20. Descriptive statistics were calculated and reported as frequencies and percentages. Chi-square (*χ*^2^) tests were used to assess associations between IPI prevalence and sociodemographic and potential risk factors. Logistic regression analyses were conducted to determine the strength of these associations. Multivariate logistic regression was employed to identify the most significant predictors of IPIs. Variables with *p* values < 0.25 in univariate analyses were included in multivariate models. Variables with *p* values < 0.05 at a 95% confidence interval were considered statistically significant.

### 2.9. Ethical Considerations and Study Conduct

The study received ethical approval from the Research Ethics Review Committee of the College of Natural and Computational Sciences, University of Gondar (Ref. No. CNCS/02/03/558/2023). Additionally, permission was obtained from Kidus Harvey Health Center. All participants voluntarily agreed to participate after receiving a detailed explanation of the study's objectives. Their personal information was treated with strict confidentiality, and each participant was assigned a unique code number. The researcher covered the costs associated with diagnosis and treatment for any positive cases.

## 3. Results

### 3.1. Sociodemographic Characteristics of the Study Participants

The study comprised a total of 404 participants, of whom 205 (50.7%) were male and 199 (49.3%) were female. In terms of age distribution, 65 (16.1%) participants were aged 5–14 years, 148 (36.6%) were aged 15–24 years, and 191 (47.3%) were aged 25 years and older. Regarding family size, 194 (48%) families had three or fewer members, 117 (29%) had four to six members, 59 (14.6%) had seven to nine members, and 34 (8.4%) had more than nine members (Table 1).

### 3.2. Prevalence of IPIs

**Figure 1:**
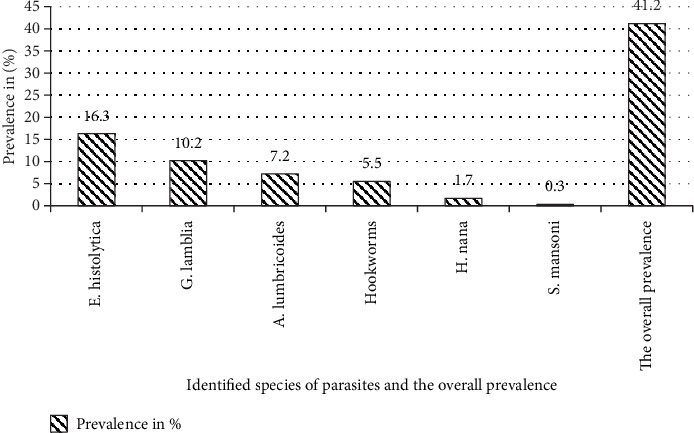
Prevalence of identified intestinal parasite species and the overall prevalence.

As Figure 1 shows, the overall prevalence of IPIs was 41.09% (166/404). Females were more likely to experience multiple infections than males (61.5% vs. 38.5%). The 25–34 age group exhibited the highest prevalence of multiple parasite infections (76.9%), followed by the 15–24 age group (23.1%). A total of six distinct intestinal parasite species were identified in this study: two protozoan and four helminths species. The *Entamoeba histolytica/dispar* emerged as the most prevalent parasite, accounting for 16.3% (66/404) of cases, followed by *Giardia lamblia* (10.2%, 41/404). Multiple infections were observed in 13 participants. *Entamoeba histolytica* was implicated in most cases, with *E. histolytica* and *Giardia lamblia* being the most common combination, accounting for 53.8% of all multiple infections (*n* = 7/13). Triple infections were rare, with only one case detected (Figure 1).

### 3.3. Sociodemographic Factors Associated With IPIs

Analysis of sociodemographic factors revealed significant associations with parasite infection. Married participants were twice as likely to be infected compared to single individuals (COR = 1.900, CI = 1.230–2.935, *p* = 0.004). Widowed participants had a fivefold higher risk of infection than single individuals (COR = 5.067, CI = 0.896–28.639, *p* = 0.004). Regarding education, illiterate participants were more susceptible to parasite infection than those with a diploma or higher (COR = 1.533, CI = 0.197–1.952, *p* = 0.037^∗^). Concerning occupational status, farmers were four times more likely to be infected compared to government employees (COR = 3.671, CI = 1.500–9.428, *p* = 0.005), and housewives had a significantly higher risk (COR = 2.644, CI = 1.029–6.796, *p* = 0.043). In relation to family size, participants with more than nine family members were three times more likely to be infected compared to those with three or fewer family members (COR = 3.359, CI = 1.580–7.140, *p* = 0.002) (Table 2).

### 3.4. Factors Related to Lifestyle Associated With IPI

Table 3 reveals significant associations between certain behaviors and the risk of infection. Participants who washed their hands before eating only sometimes were twice as likely to be infected as those who always washed their hands (COR = 2.022, CI = 1.257–3.253, *p* = 0.004^∗^). Additionally, individuals who had contact with soil demonstrated a higher incidence of infection (COR = 1.708, CI = 0.465–1.977, *p* = 0.006^∗^). Finally, eating raw meat was also linked to an increased risk of infection, with participants engaging in this behavior being approximately twice as likely to be infected as those who avoided it (COR = 1.91, CI = 0.388–2.900, *p* = 0.014^∗^) (Table 3).

### 3.5. Multivariate Logistic Regression Analysis of Selected Variables

Multivariate logistic regression analysis revealed significant associations between several sociodemographic factors and the prevalence of IPIs. Specifically, marital status, occupational status, family size, handwashing practices, shoe-wearing habits, contact with water bodies, source of drinking water, and consumption of raw meat were all identified as significant predictors of IPI (*p* < 0.05). Among the study participants, married individuals exhibited a threefold increased risk of IPI compared to their single counterparts (AOR = 3.536, CI = 1.515–8.250, *p* = 0.003). Furthermore, farmers were found to be more than twice as likely to be infected with intestinal parasites as government employees (AOR = 2.4647, CI = 0.816–7.337, *p* = 0.040). Regarding hygiene practices, participants who occasionally wore shoes were approximately twice as likely to be infected with intestinal parasites compared to those who consistently wore shoes (AOR = 1.871, CI = 0.292–2.594, *p* = 0.044). Additionally, individuals who consumed raw meat demonstrated a higher likelihood of intestinal parasite infection compared to those who refrained from this practice (AOR = 1.52, CI = 0.187–3.664, *p* = 0.001) (Table 4).

## 4. Discussion

IPIs represent a significant public health burden in Ethiopia, as in many developing nations. In this study, the overall prevalence of IPIs among the study participants was 41.09%. Previous studies conducted in Shahura Health Center, Ethiopia (56.9%) [2], and Teda Health Center, Ethiopia (62.2%) [15] reported a higher overall prevalence rates compared to the present investigation. These discrepancies may be attributed to variations in study periods, socioeconomic conditions, climatic factors, environmental sanitation practices, and geographic characteristics across the study regions.

In the present study, *E. histolytica/dispar* was identified as the dominant cause of IPIs, and this was consistent with findings from the Shahura Health Center [2]. The second most prevalent IPI was *G. lamblia* (10.2%), and it is higher than the reported 9% prevalence in the East Wollega Zone of the Chelaleki Health Center, Ethiopia [16]. Conversely, the prevalence observed in the present study was lower than the 23.7% and 32.7% rates previously documented in the Bereka Medical Center, Ethiopia [17], and Axum St. Marry hospital, Ethiopia, respectively [18]. The predominance of protozoan parasites in the study area may be attributed to several factors, including contaminated water sources, poor food handling practices, inadequate hand hygiene before and after meals, and low latrine coverage.

The prevalence of *A. lumbricoides* in this study (7.2%) was higher than that reported in a previous investigation at the Jimma Health Center, Ethiopia (5.7%) [19]. However, it was significantly lower than the prevalence rates observed in Delgi, Ethiopia (48%) [20], and among residents of Jimma Town, Ethiopia (27.6%) [21]. These differences may be attributed to variation in geographical factors, waste management practices, soil contact behaviors, and the contact frequency of contaminated hand with mouth. Furthermore, the present study revealed a considerable overall prevalence of soil-transmitted helminth infections, i.e., *A. lumbricoides* and hookworm species. These findings can be attributed to several factors, such as limited shoe-wearing habits, which increase the risk of hookworm infection, and inadequate hand hygiene practices before eating, combined with frequent soil contact, which elevates the risk of *A. lumbricoides* and *H. nana* infections.

Multiple parasitic infections were also observed in the current study, with 3% and 0.2% of participants exhibiting double and triple infections, respectively. The prevalence of multiple infections in this study was lower than that reported in a previous investigation at the Bereka Medical Center, Ethiopia (5.6%) [17]. This discrepancy may be due to variations in environmental contamination levels, socioeconomic factors, and levels of awareness.

Sociodemographic characteristics and associated factors contribute a lot for the distribution of IPIs. The determinant factors of IPI in this study subjects were marital status, occupational status, large family size, frequency of handwashing before food, frequency of shoe wearing, habits of contact with water bodies, source of water from stream, personal hygiene, and habitats of eating raw meat. In line with this, the participants who sometimes wash their hands before meal were more likely to be infected than who always washed (AOR = 1.625, CI = 0.416–3.613, *p* = 0.043). This may be due to lack of awareness about the exposure of eating food without washing hands to IPI. Married participants were over three times more likely to be infected than single individuals (AOR = 3.536, CI = 1.515–8.250, *p* = 0.003). Potential factors contributing to this increased risk among married individuals may include shared living spaces (e.g., bathrooms, kitchens), increased social interactions, and potential changes in hygiene practices.

Participants who consumed raw meat were more as likely to be infected with intestinal parasites compared to those who did not (AOR = 1.52, CI = 0.187–0.664, *p* = 0.001). This finding aligns with established knowledge linking raw meat consumption to parasite exposure and infection. Occupational factors also influenced infection risk, with farmers demonstrating a more than twofold increased likelihood of parasitic infection compared to government workers (AOR = 2.447, CI = 0.816–7.337, *p* = 0.040). Farmers' close proximity to animals and potential exposure to contaminated environments, including animal waste and soil, increase their vulnerability to intestinal parasites. Additionally, their risk is aggravated by limited access to clean water, frequent contact with contaminated soil and irrigation water, poor sanitation facilities, and improper hygiene practices. Concerning family size, individuals residing in households with more than nine members were nearly three times as likely to be infected compared to those in smaller families with fewer than three members (AOR = 2.860, CI = 0.619–13.206, *p* = 0.008). This finding may be attributed to the increased density of living arrangements in larger families, leading to greater exposure to pathogens through shared household items and frequent contact with a larger number of individuals.

## 5. Conclusions

This study revealed a high prevalence of IPIs among individuals attending Kidus Harvey Health Center, indicating a significant public health concern. The predominant parasitic species identified were *E. histolytica/dispar*, *G. lamblia*, and *A. lumbricoides*, with multiple parasitic infections frequently observed. Significant associations with IPIs included infrequent handwashing before meals, infrequent shoe wearing, frequent contact with water bodies, consumption of untreated water sources, lower socioeconomic status, improper latrine use, and raw meat consumption, likely due to inadequate food preparation. Married and widowed individuals exhibited higher vulnerability, possibly due to shared living environments. To mitigate these risks, Kidus Harvey Health Center should implement mandatory hygiene education sessions focusing on handwashing, shoe wearing, and safe food preparation. A regular deworming schedule targeting high-risk groups, including married/widowed individuals and those with low socioeconomic status, is crucial. Targeted counseling for patients reporting infrequent latrine use, raw meat consumption, or use of nonpiped water sources and collaboration with health extension workers for home visits will further enhance preventive measures.

## Figures and Tables

**Table 1:** Sociodemographic characteristics of study participants in Kidus Harvey Health Center, Ayna Bugina District, North Wollo, Ethiopia.

**Characteristics**	**Categories**	**Frequency (** **n** **)**	**Percentage (%)**
Sex	Male	205	50.7
	Female	199	49.3

Age group (in years)	5–14	65	16.1
	15–24	148	36.6
	≥ 25	191	47.3

Residence	Urban	129	31.9
	Rural	275	68.1

Marital status	Single	159	39.4
	Married	224	55.4
	Divorced	15	3.7
	Widowed	6	1.5

Education level	Illiterate	136	33.7
	Primary school	167	41.3
	Secondary school	59	14.6
	Diploma and above	42	10.4

Occupational status	Government employee	37	9.2
	Merchant	77	19.2
	Farmer	92	22.8
	Housewife	76	18.8
	Student	122	30.2

Family size	≤ 3	194	48
	4–6	117	29
	7–9	59	14.6
	> 9	34	8.4

Monthly income	< 1500 birr	254	62.9
	1500–3000 birr	93	23
	> 3000 birr	56	13.9

**Table 2:** Univariate logistic regression analysis for sociodemographic-related factors associated with IPIs among study subjects attending the Kidus Harvey Health Center.

**Factors**	**Categories**	**N** ** (%)**	**IPIs**		**COR, (95% CI), ** **p** ** value**
			**Positive (%)**	**Negative (%)**	
Sex	Male	205 (50.7)	76 (37.1)	129 (62.9)	1
	Female	199 (49.3)	74 (37.2)	125 (62.8)	1.005, 0.671–1.505, 0.981

Age	5–14	65 (16.1)	20 (30.8)	45 (69.2)	1
	15–24	148 (36.6)	55 (37.2)	93 (62.8)	1.331, 0.713–2.482, 0.369
	≥ 25	191 (47.3)	75 (39.3)	116 (60.7)	1.455, 0.797–2.655, 0.222

Residency	Urban	129 (31.9)	50 (38.8)	79 (61.2)	1
	Rural	275 (68.1)	100 (36.4)	175 (63.6)	0.903, 0.587–1.389, 0.642

Marital status	Single	159 (39.4)	45 (28.3)	114 (71.7)	1
	Married	224 (55.4)	96 (42.9)	128 (57.1)	1.900, 1.230–2.935, 0.004⁣^∗^
	Divorced	15 (3.7)	5 (33.3)	10 (66.7)	1.267, 0.410–3.912, 0.681
	Widowed	6 (1.5)	4 (66.7)	2 (33.3)	5.067,0.896–28.639, 0.004⁣^∗^

Education level	Illiterate	136 (33.7)	68 (50)	68 (50.0)	1.533, 0.197–1.952, 0.037⁣^∗^
	Primary school	167 (41.3)	58 (34.7)	109 (65.3)	0.737, 0.463–1.176, 0.201
	Secondary School	59 (14.6)	20 (33.4)	39 (66.6)	1.019, 0.549–1.892, 0.952
	≥ Diploma	42 (10.4)	4 (9.5)	36 (90.5)	1

Occupational status	Gov. employee	37 (9.2)	7 (18.9)	30 (81.1)	1
	Merchant	77 (19.1)	30 (39)	47 (61)	2.736, 1.067–7.014, 0.056
	Farmer	92 (22.8)	43 (46.7)	49 (53.3)	3.761, 1.500–9.428, 0.005⁣^∗^
	Housewife	76 (18.8)	29 (38.2)	47 (61.8)	2.644, 1.029–6.796, 0.043⁣^∗^
	Student	122 (30.2)	41 (33.6)	81 (66.4)	2.169, 0.878–5.359, 0.093

Family size	< 3	194 (48)	63 (32.5)	131 (67.5)	1
	4–6	117 (29)	50 (42.7)	67 (57.3)	1.552, 0.966–2.492, 0.069
	7–9	59 (14.6)	16 (27.1)	43 (72.9)	0.774, 0.405–1.479, 0.438
	> 9	34 (8.4)	21 (61.8)	13 (38.2)	3.359, 1.580–7.140, 0.002⁣^∗^

Monthly income	< 1500 birr	254 (63)	108 (42.5)	146 (57.5)	1.627, 0.740–2.382, 0.043⁣^∗^
	1500–3000 birr	93 (23.1)	29 (31.2)	64 (68.8)	0.746, 0.449–1.238, 0.257
	> 3000 birr	56 (13.9)	13 (23.6)	43 (76.4)	1

*Note:* 1 = reference, *N* = total number of study participants.

Abbreviation: COR = crude odds ratio.

⁣^∗^Statistically significant at *p* < 0.05.

**Table 3:** Univariate logistic regression analysis of factors associated with personal life style and hygiene with IPIs among study subjects attending Kidus Harvey Health Center.

**Risk factors**	**Categories**	**N** ** (%)**	**IPIs**		**COR, (95% CI), ** **p** ** value**
			**Positive (%)**	**Negative (%)**	
Handwashing before food	Yes	324 (80.2)	122 (37.7)	202 (62.3)	1
	No	80 (19.8)	28 (35)	52 (65)	0.892, 0.535–1.487, 0.660

Frequency of handwashing before food	Always	217 (67.2)	70 (32.3)	147 (67.7)	1
	Sometimes	106 (32.8)	52 (49.1)	54 (50.9)	2.022, 1.257–3.253, 0.004⁣^∗^

Shoe-wearing habit	Yes	355 (87.6)	128 (36.2)	226 (63.8)	1
	No	49 (12.1)	22 (44.9)	27 (55.1)	1.445, 0.791–2.641, 0.232

Frequency of shoe wearing	Always	265 (74.2)	88 (33.2)	177 (66.8)	1
	Sometimes	92 (25.8)	42 (45.7)	50 (54.3)	1.690, 1.042–2.740, 0.033⁣^∗^

Habit of soil contact	Yes	246 (60.9)	99 (40.2)	147 (59.8)	1.708, 0.465–1.977, 0.006⁣^∗^
	No	158 (39.1)	51 (32.3)	107 (67.7)	1

Dirty material under finger nail	Yes	183 (45.3)	74 (40.4)	109 (59.6)	1
	No	221 (54.7)	76 (34.4)	145 (65.6)	0.772, 0.515–1.158, 0.211

Presence of latrine at home	Yes	283 (70.2)	100 (35.3)	183 (64.7)	1
	No	121 (29.8)	49 (40.8)	71 (59.2)	0.792, 0.511–1.227, 0.296

Frequency of latrine use	Always	134 (47.3)	44 (32.8)	90 (67.2)	1
	Sometimes	149 (52.7)	56 (37.6)	93 (6.4)	1.52, 0.755–2.010, 0.004

Personal hygiene	Good	178 (44.7)	70 (39.3)	108 (60.7)	1
	Poor	226 (55.3)	80 (34.5)	146 (65.5)	0.814, 0.541–1.226, 0.325

Contact with water bodies	Yes	228 (56.4)	97 (42.5)	131 (57.5)	0.582, 0.384–0.882, 0.311
	No	176 (43.6)	53 (30.1)	123 (69.9)	1

Reason for water contact	Swimming	35 (15.1)	11 (31.4)	24 (68.6)	1
	Washing clothes	62 (26.7)	20 (32.3)	42 (67.7)	1.039, 0.426–2.531, 0.933
	Irrigation	45 (19.4)	24 (53.3)	21 (46.7)	2.494, 0.991–6.277, 0.042
	Showering	90 (38.8)	43 (47.8)	47 (52.2)	1.996, 0.875–4.554, 0.100

Handwashing after toilet	Yes	152 (37.6)	49 (32.2)	103 (67.8)	1
	No	252 (62.4)	101 (40.1)	151 (59.9)	1.406, 0.921–2.147, 0.115

Frequency of handwashing after toilet	Always	121 (78.6)	40 (33.1)	81 (66.9)	1
	Sometimes	33 (21.4)	10 (30.3)	23 (69.7)	0.880, 0.383–2.026, 0.765

Source of drink water	Tanker water	17 (4.2)	9 (52.9)	8 (47.1)	1
	Well water	81 (20)	30 (37)	51 (63)	0.523, 0.182–1.500, 0.228
	Stream water	152 (37.6)	67 (44.1)	85 (55.9)	1.56, 0.129–2.981, 0.046⁣^∗^
	Pipe water	154 (38.1)	44 (28.6)	110 (71.4)	0.701, 0.257–1.914,0.488

Eating unwashed vegetable	Yes	114 (28)	50 (44.2)	63 (55.8)	1
	No	290 (72)	100 (34.5)	190 (65.5)	0.663, 0.426–1.033, 0.069

Eating raw meat	Yes	143 (35.1)	64 (45.4)	77 (54.6)	1.91, 0.388–2.900, 0.014⁣^∗^
	No	261 (64.9)	86 (33)	175 (67)	1

*Note:* 1 = reference, *N* = total number of study participants.

Abbreviation: COR = crude odds ratio.

⁣^∗^Statistically significant at *p* < 0.05.

**Table 4:** Multivariate logistic regression analysis of selected factors associated with IPI among study subjects attending Kidus Harvey Health Center.

**Factors**	**Categories**	**N** ** (%)**	**IPIs**		**AOR, (95% CI), ** **p** ** value**
			**Positive (%)**	**Negative (%)**	
Marital status	Single	159 (39.4)	45 (28.3)	114 (71.7)	1
	Married	224 (55.4)	96 (42.9)	128 (57.1)	3.536, 1.515–8.250, 0.003⁣^∗^
	Divorced	15 (3.7)	5 (33.3)	10 (66.7)	3.623, 0.670–19.580, 0.135
	Widowed	6 (1.5)	4 (66.7)	2 (33.3)	5.487, 0.565–53.277, 0.142

Occupational status	Gov. employee	37 (9.2)	7 (18.9)	30 (81.1)	1
	Merchant	77 (19.1)	30 (39)	47 (61)	1.549, 0.899–7.225, 0.078
	Farmer	92 (22.8)	43 (46.7)	49 (53.3)	2.447, 0.816–7.337, 0.040⁣^∗^
	Housewife	76 (18.8)	29 (38.2)	47 (61.8)	1.232, 0.371–4.098, 0.733
	Student	122 (30.2)	41 (33.6)	81 (66.4)	1.988, 0.965–9.253, 0.058

Family size	≤ 3	194 (48)	63 (32.5)	131 (67.5)	1
	4–6	117 (29)	50 (42.7)	67 (57.3)	1.010, 0.442–2.307, 0.980
	7–9	59 (14.6)	16 (27.1)	43 (72.9)	0.448, 0.162–1.243, 0.123
	> 9	34 (8.4)	21 (61.8)	13 (38.2)	2.860, 0.619–13.206, 0.008⁣^∗^

Frequency of handwashing before food	Always	217 (67.2)	70 (32.3)	147 (67.7)	1
	Sometimes	106 (32.8)	52 (49.1)	54 (50.9)	1.625, 0.416–3.613, 0.043⁣^∗^

Frequency of shoe wearing	Always	265 (74.2)	88 (33.2)	177 (66.8)	1
	Sometimes	92 (25.8)	42 (45.7)	50 (54.3)	1.871, 0.292–2.594, 0.044⁣^∗^

Contact with water bodies	Yes	228 (56.4)	97 (42.5)	131 (57.5)	1.735, 0.400–2.350, 0.022⁣^∗^
	No	176 (43.6)	53 (30.1)	123 (69.9)	1

Source of drink water	Tanker water	17 (4.2)	9 (52.9)	8 (47.1)	1
	Well water	81 (20)	30 (37)	51 (63)	0.367, 0.051–2.612,
	Stream water	152 (37.6)	67 (44.1)	85 (55.9)	0.507, 0.056–1.685, 0.044⁣^∗^
	Pipe water	154 (38.1)	44 (28.6)	110 (71.4)	0.247, 0.048–1.275, 0.095

Eating raw meet	Yes	143 (35.1)	64 (45.4)	77 (54.6)	1.52, 0.187–3.664, 0.001⁣^∗^
	No	261 (64.9)	86 (33)	175 (67)	1

*Note:* 1 = reference, *N* = total number of study participants.

Abbreviation: AOR = adjusted odds ratio.

⁣^∗^Statistically significant at *p* < 0.05.

## Data Availability

The entire raw data of this study are available in the hands of the authors and will be submitted for reasonable requests.

## General Statement

*Statement of the Guidelines*. In this study, all tasks (including questionnaire administration, stool sample collection, and diagnosis) were carried out in accordance with relevant WHO intestinal parasite diagnosis guidelines and regulations.
